# TSPO as a target for treatments of diseases, including neuropathological disorders

**DOI:** 10.1038/cddis.2015.294

**Published:** 2015-10-15

**Authors:** L Veenman, A Vainshtein, M Gavish

**Affiliations:** 1 Department of Neuroscience, Faculty of Medicine, Rappaport Family Institute for Research in the Medical Sciences, Technion – Israel Institute of Technology, Haifa, Israel

Recent knockout studies in mice have heightened interest in the 18-kDa translocator protein (TSPO) as a target for drug treatment of various diseases.^1^ Although TSPO function may be inconspicuous in health, various studies have suggested TSPO may be relevant for responses to stress, disease, and injury.^2, 3^ In addition, TSPO was found necessary for preimplantation embryo development.^4^ Recent studies have elucidated the molecular structure of TSPO and interactions with its ligands.^1^ Over the past decennia, several groups have developed various ligands interacting with the TSPO.^5^ Recently, it was found that relatively small (~400 molecular weight) tricyclic compounds based on the bicyclic quinazoline as a scaffold can be used to regulate the functions of the TSPO.^6^

TSPO can be found throughout the body.^2^ Moderate TSPO expression also occurs in healthy central nervous system, which can increase in association with disease and injury, both in glia and in neurons.^7, 8^ The TSPO takes part in various cellular functions, for example, regulation of cell death and expression of numerous genes. Associated with these functions, the primary locations of the TSPO include mitochondria and perinuclear sites.^9^ Since a few years, TSPO function in cell differentiation is becoming more appreciated.^6^ Embryonic neuronal development includes migration of enormous numbers of progenitor cells into specific target areas. Subsequently, millions of these cells reaching the target area die off, while only a very restricted number of progenitor cells (a few ten thousands) develop into mature neurons.^10^ Embryonic development of brain includes maintenance of a glial scaffold to support developing neurons. The interactions with glia, including radial glia, appear to contribute to the neurodifferentiation of progenitor cells.^11^ Regarding trauma of healthy mature brain tissue, programmed cell death can result from various mechanic and toxic insults and also disease, including expanding brain cancer. In adults of many species of vertebrates, migration of progenitor cells into damaged brain areas can allow for replenishment of depleted neurons.^12^ Regarding brain cancer, glioma cells are almost per definition not differentiated and resistant to programmed cell death. At the same time, they induce cell death in surrounding healthy tissue, for example, by excessive glutamate release, as well as by mechanic compression of the brain in its enclosure of the skull.^13^ It appears that tricyclic compounds based on a quinazoline scaffold can induce effects in cell culture that mimic those of embryonic brain development.^6^ This includes: (1) protecting cells of glial origin from cell death, (2) stimulating massive cell death of neuronal progenitor cells as induced by glutamate, and (3) stimulating extensive neuronal differentiation of the surviving neuronal progenitor cells (Figure 1).

To discern whether these cell culture findings may actually translate to implications for the whole organism, we used established mammalian models for progressing brain damage and neurodegeneration for application of the classical TSPO ligand PK 11195 and the quinazoline-based tricyclic compounds. These animal models included systemic kainic acid injections in rats to induce excitotoxic damage in hippocampus, amygdala, and pyriform cortex and subsequent edemic damage spread throughout the brain.^6, 14^ We also studied the R6-2 transgenic mouse model for the human hereditary neurodegenerative disease of Huntington, which presents damage in the striatum and cortex, motor disturbances, and finally, early death.^6, 15^ In general, TSPO ligands, and especially the quinazoline-derived tricyclic compounds, are not only very effective regarding cell protection in cell culture but also are involved in neuroprotection and neuro-repair following brain damage owing to neurotoxicity and a hereditary neurodegenerative disease. These compounds prevent the emergence of seizures, attenuate the hyperreactivity in response to handling that typically occurs in the days and weeks after the seizures, and attenuates occurrence of damage in the hippocampus, pyriform cortex, and amygdala owing to kainic acid. Chronically used to treat the after-effects of kainic acid injections, curative and restorative effects of the quinazoline-based compounds were observed. Notably, these compounds attenuated hyperactivity in response to handling, which is considered to be a consequence of edema developing throughout the brain after excitotoxic damage.^6^ Surprisingly, PK 11195 did not work at all in the animal model for Huntington disease (R6-2 transgenic mice). However, several of the quinazoline-derived tricyclic compounds could reduce locomotor aberrations and also increase the lifespan of R6-2 mice.^6^ The treatments were daily injections for the entire lifespan of these R6-2 mice. These compounds appeared to preclude heart failure, which otherwise occurs typically in R6-2 mice and also in Huntington disease patients.

As the molecules expanded from a bicyclic quinazoline scaffold were designed to bind to the TSPO and to regulate TSPO-related functions, we assume that this is at least part of the way by which they regulate neurodifferentiation. It was also found that the classical TSPO ligand PK 11195 can induce neurodifferentiation. In this context, it is known that TSPO regulates several functions, including but not restricted to: (1) programmed cell death, and 2) gene expression for various proteins essential for neurodifferentiation, such as growth factors and their receptors; as well as proteins related to adhesion, migration, cell cycle, and so on.^5^

To summarize: in cell culture, specific tricyclic quinazoline derivatives and classical TSPO ligands can regulate programmed cell death and induce neurodifferentiation. In animal models for brain injury and disease, these compounds prevent acute damage and behavioral disorders. Applied chronically during the period of progressing damage due to injury and disease, specific tricyclic quinazoline derivatives targeting the TSPO show curative effects regarding brain damage, behavior, and life expectancy. Thus these tricyclic molecules by themselves can induce diverse processes that also occur during normal embryonic brain development and promote protective and restorative responses in adult brain tissue damage (Figure 1). Importantly, even with lifelong chronic treatments with tricyclic quinazoline derivatives,^6^ no adverse effects were discerned.

## Figures and Tables

**Figure 1:**
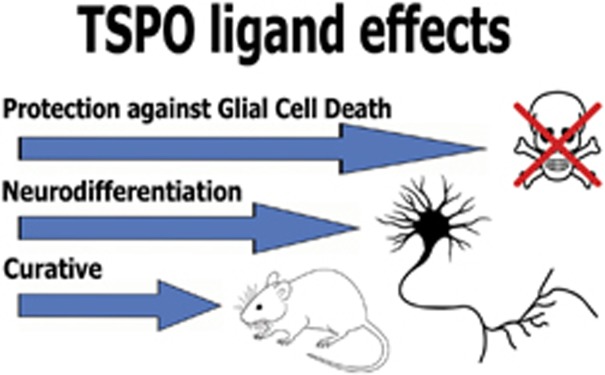
Beneficial effects of TSPO ligands regarding brain disease and brain damage. TSPO ligands protect glioma cells in culture from cell death induced by glutamate. TSPO ligands induce neurodifferentiation of PC12 cells. TSPO ligands prevent seizures, behavioral disorders, and brain damage
